# Lead Colic

**Published:** 1870-12

**Authors:** 


### LEAD COLIC

Is a disease to which painters, and workers in red and white lead, are subject, causing severe pains, tedious sickness, and often death. The disease is partially owing perhaps to breathing the fumes, but mainly from particles taken into the stomach by the food which is handled. Workmen can effect a total exemption from the disease by attending rigidly to three things.

*First.* Keep the finger nails trimmed closely so as to prevent particles of lead from collecting under them and transference to the bread in eating it.

*Second.* Wash the hands well with soap and water, and rinse the mouth before eating.

*Third.* Drink half a pint of sweet milk at each meal to antagonize the influence of any particles of lead which may find their way into the stomach. It has been found in thousands of cases that an habitual attention to these things secures an entire exemption from lead colic.

